# Suppression of hyaluronidase reduces invasion and establishment of *Haemonchus contortus* larvae in sheep

**DOI:** 10.1186/s13567-020-00831-8

**Published:** 2020-08-27

**Authors:** Xiangshu Yang, Sawar Khan, Xiaochao Zhao, Jiayan Zhang, Ayesha Nisar, Xingang Feng

**Affiliations:** 1 Shanghai Veterinary Research Institute, Chinese Academy of Agricultural Sciences, Key Laboratory of Animal Parasitology, Ministry of Agriculture of China, Shanghai, 200241 People’s Republic of China; 2 College of Life Sciences, Shanghai Normal University, Shanghai, 200234 People’s Republic of China

**Keywords:** *Haemonchus contortus*, hyaluronidase, RNA interference, virulence factor, ex vivo tissue explants, in vivo

## Abstract

*Haemonchus contortus* is a hematophagous endoparasite of small ruminants, which is responsible for huge economic losses in livestock sector. Hyaluronidase produced by infective larvae of *H. contortus* can degrade hyaluronic acid present in the host’s abomasal tissue. Thus, it facilitates larval tissue invasion and early establishment. We herein explored this ability of hyaluronidase in *H. contortus*, and tested whether hyaluronidase is utilized as a virulence factor by *H. contortus* while establishing the infection. We first successfully blocked the hyaluronidase gene in L3 larvae by RNA interference (RNAi), which was subsequently confirmed by qPCR, enzymatic activity, and immunohistochemistry assays. Using these larvae we then conducted in vivo and in vitro assays on sheep to assess the effects of hyaluronidase suppression on larval invasion and establishment of infection. The in vivo assay showed a significant drop in worm burden in siRNA treated group in comparison to control group. During in vitro assay we applied an ovine ex vivo model where siRNA treated group of larvae showed significantly reduced invasion of the abomasal tissue explants as compared to control group. These findings indicate that hyaluronidase plays a key role in host’s tissue invasion and larval establishment, and it is used as a virulence factor by *H. contortus* while establishing the infection. As an invasive virulence molecule, its functional research is thus conducive to the prevention of haemonchosis.

## Introduction

*Haemonchus contortus* is an important gastrointestinal parasitic nematode of domestic and wild small ruminants whose origin is traced back to an assemblage of antelopes in Africa during the late Tertiary period [1–3]. It feeds on blood while living in the host’s abomasum and causes an infection called haemonchosis (a pathological condition characterized by anaemia, weight loss, and even mortality in infected animals), thereby leading to serious economic losses [4–6]. Currently, the prevention and control of *H. contortus* is based on application of drugs such as macrocyclic lactones, salicylanilides, and benzimidazoles. Recently, a vaccine named Barbervax^®^ (http://barbervax.com.au/) was also launched as alternative to chemical control of *H. contortus*. This parasite has shown a great ability to develop resistance against all anthelmintic drugs [7–11], and even showed to develop resistance within a few years of application of a new drug [12–15]. It has emerged as a model parasitic species to serve as a system for functional and comparative genomics to test the anthelmintic resistance, and drug and vaccine discovery efforts as alternative methods of control [16–18]. Its use as a model is largely due to its rapid ability to acquire drug resistance, the relative amenability to experimentation under lab conditions [16], the development of extensive genomic resources [19, 20], testing novel functional validation approaches, such as RNAi [21, 22], and its closer phylogeny with other nematodes of clade V [23]. It has a successful track record in anthelmintic resistance [24] and drug discovery [25].

*Haemonchus contortus* undergoes both, the free living as well as the parasitic stages of its life cycle. The eggs hatch into free living larval stage L1, which is followed by another free living stage L2. The L2 then matures into infective stage L3. These infective larvae (L3) are ingested by host. Once they reach into the abomasum of the host they start to invade into the mucosa and develop into L4. These final stage larvae (L4) then molt into adult worms. Invasion into the mucosa is a crucial step in larval establishment of *H. contortus*, and transition from a free-living to a parasitic life style takes place at this stage [26]. Thus, it could be a putative site for exploring new preventive and control strategies for haemonchosis. However, not much is known about the mechanism and molecular mediators involved in larval establishment at this step.

Hyaluronidases are a family of enzymes that degrade hyaluronic acid [27, 28]. Hyaluronic acid is an anionic, nonsulfated polysaccharide (consisting on glycosaminoglycan), which constitutes the intercellular ground substance of connective tissue, controls tissue permeation, and maintains the integrity of extracellular matrix [29, 30]. Hyaluronidases are involved in physiological as well as in pathological activities. They play a critical role in: cell differentiation, proliferation, and adhesion [31]; embryogenesis [32]; osteogenesis and skeletal growth [33, 34]; and wound healing and tissue repair [35, 36]. Bacteria, leeches, and venomous animals (in venom) use hyaluronidase to promote invasion and spread through the tissue by destroying its integrity [36]. In parasitic nematodes the hyaluronidases are postulated to act as a virulence factor while facilitating larval invasion and migration through the host’s tissues [37, 38].

Hyaluronic acid is also present abundantly in the abomasal mucosa (interstitial connective tissue) of the *H. contortus*’ hosts, and a temporal expression of the hyaluronidase has been reported during L3 to L4 developmental process in *H. contortus* [39]. In this context the hyaluronidase of *H. contortus*, thus, might act as the virulence factor while facilitating the invasion of L3 into the mucosal lining of host’s abomasum, protecting larvae from elimination, and providing larvae with immediate access to nutrients. However, it has not been proven so far in *H. contortus*. We therefore in this study tested the question whether hyaluronidase could be a virulence factor that assists the *H. contortus* to invade and colonize the host’s abomasal tissue. To this end, we first blocked the hyaluronidase gene in L3 larvae by RNA interference (RNAi). We then used these larvae to perform in vivo and in vitro assays to monitor the ability of larvae to cause infection. Finally, our results showed that hyaluronidase enhances the larval invasion of the abomasal tissue during *H. contortus* infection.

## Materials and methods

### Parasites and animals

Parasite free sheep (15 animals of a local breed) used in this study were purchased from a local farm in Shanghai. Sheep were 3 months old and were under 15 kg of body weight. Feces were inspected continuously for 3 weeks for parasitic eggs in order to ascertain that sheep were parasite free. Furthermore, it was also ensured that the drinking water and feed were parasite free. Six to eight weeks old BALB/c mice were purchased from the Shanghai Experimental Animal Center, Chinese Academy of Sciences. The *H. contortus* L3 larvae of an Australian strain that we have been maintaining in sheep for several years [6, 40], were harvested from faecal cultures.

### RNA interference

In order to block the *H. contortus* hyaluronidase gene [(the only hyaluronidase gene sequence in *H. contortus* to date that we found under the GenBank accession number: CDJ92372.1), (% amino acid identity with other nematodes, Additional file 1)], a specific double stranded siRNA sequence (HAase-siRNA) was designed using GenScript siRNA Target Finder tool (https://www.genscript.com/tools/sirna-target-finder). As a negative control, a non-specific double stranded siRNA sequence (NC-siRNA) was also designed that was not targeting any of *H. contortus* genes. These sequences were crossed checked for their off targets by BLAST tool (https://parasite.wormbase.org/Haemonchus_contortus_prjeb506/Tools/Blast). All the siRNA sequences (Additional file 2) were commercially synthesized from the Gene Pharma, Shanghai. The L3 larvae were exsheathed (xL3s) with 0.2% sodium hypochlorite (for 5 min at RT), washed twice with PBS, and re-suspended into water (10,000 larvae/mL). Two groups of xL3s (HAase-siRNA, and NC-siRNA) were made for siRNA treatment. The NC-siRNA and HAase-siRNA were added to xL3s with a final concentration of 1 μg/μL. Electroporation was then applied using a Bio-Rad GenePulser (100 V for 30 ms). Both groups of xL3s were collected in a 12-well plate (about 5000/well) after electroporation, and were incubated at 37 °C, 5% CO_2_ for 48 h.

### RNA extraction and qPCR

After 48 h of culturing siRNA treated xL3s, aliquots (50 μL pellet) from both groups were subjected to total RNA extraction. Procedures were performed using QIAGEN RNeasy Mini Kit (cat#74104) as per manufacturer’s protocol. Quality and quantity of isolated RNA was checked using a Nanodrop spectrophotometer (Thermo Scientific, USA). First-strand cDNA was synthesized using 1 μg of total RNA with Takara PrimeScript RT reagent Kit having gDNA eraser (cat#RR047A) by following the manufacturer's instructions. The qPCR assays were performed with specifically designed primers (Additional file 3), and SYBR Green reagents (YEASEN Hieff^®^ cat#11202ES03) as per kit’s protocol on an ABI-7500 system in triplicate set up. Hc-NADH was used as a housekeeping/normaliser gene, to quantitate the level of HC-HAase transcript relative to it. The relative quantification of the target transcripts were performed by 2^−ΔΔCt^ method [41].

### Detection of hyaluronidase activity

Activity of hyaluronidase was assessed by 3,5-dinitrosalicylic acid (DNS) method [42]. A standard curve of hyaluronidase activity was first generated using the different concentrations of hyaluronidase (20–125 U) in serial dilutions on its substrate hyaluronic acid, HA (0.5% w/v). Briefly, 500 μL enzyme solution was mixed with 500 μL HA solution, and samples were incubated at 37 °C for 30 min. In control sample (blank) PBS was used instead of hyaluronidase. After incubation, samples were placed in boiling water for 5 min to terminate the reaction. An aliquot of 200 μL of each sample was mixed with 400 μL of DNS solution, placed in boiling water for 5 min, and loaded to a 96-well plate (150 μL/well) in triplicate. Samples were analyzed under a 540 nm wavelength of maximum absorbance with a microplate reader. Data was analyzed by linear regression (compare of slopes) and a standard curve was generated with the corresponding relationship between absorbance and concentration. Subsequently, samples (500 μL) from culture solution of siRNA treated xL3s after 48 h (from both groups of larvae) were mixed with 500 μL HA solution, and incubated at 37 °C for 30 min. Immediately after the incubation, samples were transferred to boiling water for 5 min, and an aliquot of 200 μL of each sample was taken. The aliquot was then mixed with 400 μL of DNS solution, placed in boiling water for 5 min, and loaded to a 96-well plate (150 μL/well) in triplicate. Samples were analyzed in a microplate reader under a 540 nm wavelength of maximum absorbance. The enzyme activity of the solution was calculated by already generated standard curve.

### Worm burden and morphometric analysis

A total of 15 worm-free sheep were divided into three groups (five sheep in each group). Sheep in the first group were infected with 8000 treated larvae (xL3s-HAase-siRNA) by oral feeding. The second group of sheep was infected with same numbers of xL3s-NC-siRNA larvae. Whereas the third group of sheep was left uninfected. Fecal egg count (total number of eggs per gram of feces) was conducted onward from 18 day post infection (dpi) to 32 dpi using McMaster’s technique. After 32 dpi, the sheep were slaughtered and abomasa were obtained. The adult worms recovered from abomasa were counted in each individual sheep. Worm burden was calculated as percentage of recovered adult worms in relation to total number of larvae in a given infection. Morphometric analyses were performed to capture any morphological variation that likely occurred in adult worms of any group. Body lengths were measured in adult worms (randomly chosen 50 worms) of both groups. Adults were fixed with 2.5% glutaraldehyde, and subjected to scanning electron microscopy to observe any changes occurred in body surface in both groups.

### Analysis of larval invasion of abomasal tissue explants

To observe the effects of blocking hyaluronidase on the larval invasion of abomasal tissue, an ovine ex vivo model [43, 44] was applied. Abomasal tissue obtained from a worm-free sheep (from third group) was gently washed with warm 0.85% saline, and cut down into 2 cm × 2 cm tissue pieces. A 6-well plate was used to place every tissue piece into a single well containing Hanks balance solution (added to surround but not submerge the tissues). A 5 mL syringe was cut (the needle end was removed) into a cylinder barrel (about 1.6 cm) for each sample. The syringe barrel was placed onto the center of each abomasal tissue piece into which siRNA treated xL3s (about 2400) of the respective groups were introduced. The tissue samples were then incubated at 38 °C for 3 h in the dark under high oxygen conditions. The experiments were conducted in five replicates for both groups. All operations from slaughter to incubations were performed in about 20 min. Three different tubes (50 mL centrifuge tube) were prepared for post-incubation procedures (rinse, wash, and digest). After incubation the tissues along with syringe barrel were first rinsed in the first tube containing 0.85% physiological saline to wash out all unassociated larvae. Tissues were then washed vigorously in the second tube containing 25 mL of physiological saline to further wash off the weakly attached larvae. Finally, the tissues were transferred to the third tube (containing 1% pepsin + 1% HCl) for digestion (at 38 °C for 12 h). Number of larvae in all three tubes (rinse, wash, and digest) were counted, and the percentage of larval establishment was calculated as:

$$\% \;{\text{of}}\;{\text{larval}}\;{\text{establishment}} = \left[ {{{\text{D}} \mathord{\left/ {\vphantom {{\text{D}} {\left( {{\text{R}} + {\text{W}} + {\text{D}}} \right)}}} \right. \kern-0pt} {\left( {{\text{R}} + {\text{W}} + {\text{D}}} \right)}}} \right] \times 100$$

where: D represents the number of larvae in the digest tube; R represents number of larvae in the rinse tube; and W represents number of larvae in the wash tube.

For histological analysis the tissues were fixed in 4% paraformaldehyde, processed to paraffin sections (4 μm), and subjected to H&E staining according to standard protocols. Slides were visualized and photographed using optical microscope (Nikon, model H500S).

### Primary antibody and immunohistochemistry (IHC)

The hyaluronidase gene (full-length) of *H. contortus* was PCR-amplified using specific primers: F-5′GACAAGGCCATGGCTGATATCCAACTTGACAGCTTCCCGGT3′; R-5′ACGGAGCTCGAATTCGGATCCTCAAATTAATCGGAAGTCCAGTGG3′. PCR conditions were as following. An initial pre-denaturation at 94 °C for 5 min, followed by 35 cycles of: denaturation at 94 °C for 30 s; annealing at 57 °C for 30 s; and extension at 72 °C for 1.5 min. The final extension step was of 10 min at 72 °C. PCR products were gel purified, and ligated to PET-32a vector to form a recombinant plasmid pET- 32a-Hc-Haase. The recombinant plasmid was transformed into *E. coli* BL21, and IPTG induced expression of the recombinant protein was obtained in *E. coli*. Protein was purified by nickel column, and verified on SDS page. The concentration of protein was detected by BCA (Shanghai Shenggong). The purified hyaluronidase protein was used to immunize mice by subcutaneous injection of a dose of 90 μg per week. Serum of challenged mice was collected after 5 weeks. A HRP-Goat anti-mouse universal secondary antibody (Dako, Denmark: Code K5007) was used to spot the hyaluronidase in both groups of larvae. Briefly, the samples were first processed for antigen retrieval by heating (8–15 min) in antigen retrieval buffer. Following this, endogenous peroxidase activity was blocked in 3% hydrogen peroxide for 25 min in the dark, and slides were washed with PBS. Samples were then blocked (at RT for 30 min) with 3% BSA (bovine serum albumin) prior to incubation (at 4 °C, overnight) with the primary antibody (at a dilution of 1:100). Slides were washed with PBS, and the secondary antibody (HRP-labelled) was then added. Finally, detection was performed using DAB staining (brown), while Harris hematoxylin was used as counterstain. Slides were dehydrated with ethanol and xylene, and sealed with gum. Slides were visualized and photographed using optical microscope (Nikon Eclipse 50i, model H550S).

### Statistical analysis

Statistical analyses were performed using Microsoft Excel and GraphPad Prism 6 software. A two tailed student’s *T* test was performed for determining significance changes among both groups. A p-value of < 0.05 was considered as significant. Data of enzymatic activity was analyzed by linear regression (compare of slopes) and a standard curve was generated with the corresponding relationship between absorbance and concentration.

## Results

### Silencing of hyaluronidase

**Figure 1:**
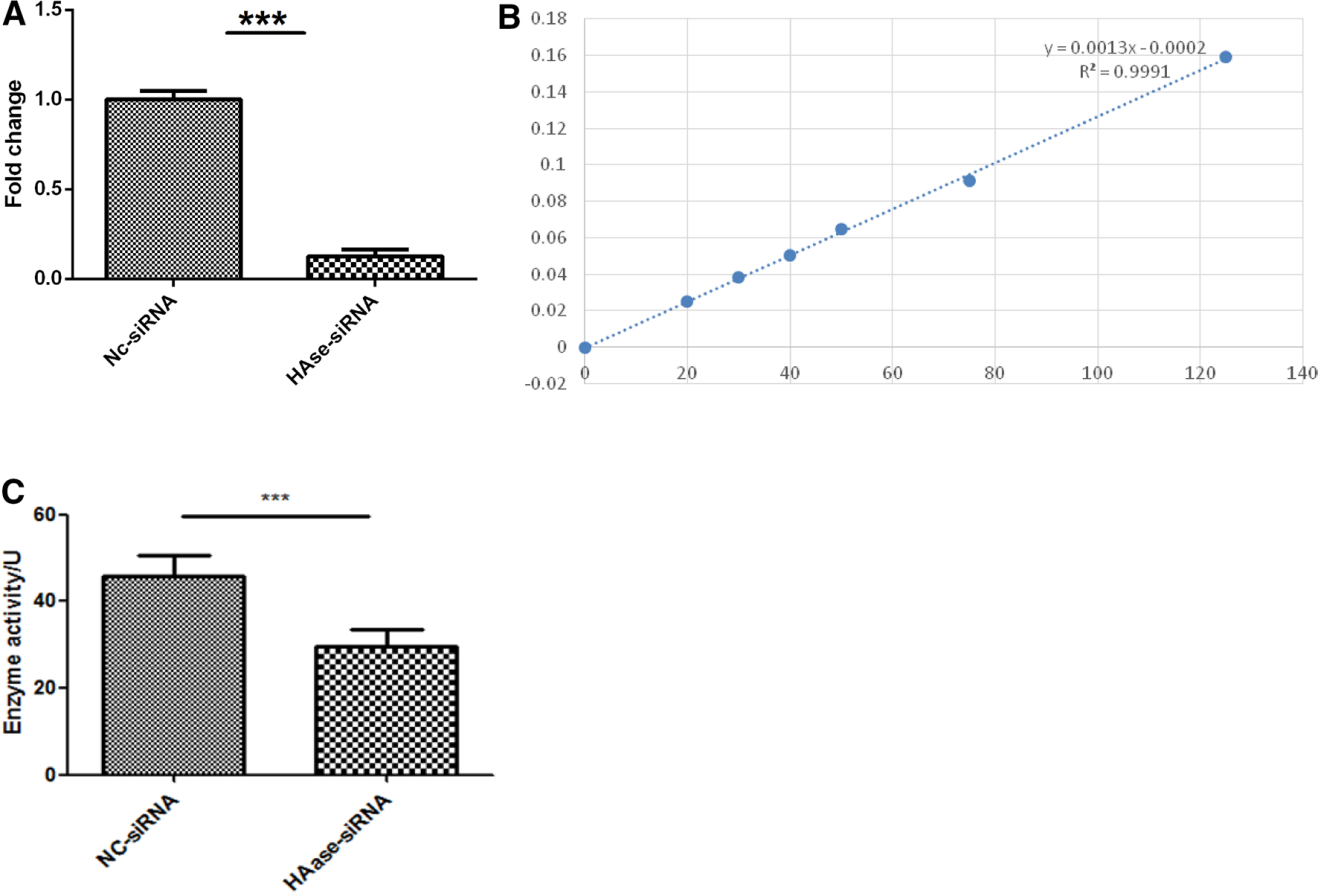
**The relative expression of hyaluronidase in** ***H. contortus*** **L3 larvae after RNAi treatment**. **A** The relative expression of hyaluronidase at transcriptional level. **B** Standard calibration curve. **C** Relative enzymatic activity of hyaluronidase after RNAi treatment. Values represent mean ± SD of three replicates. ***p < 0.0001.

We explored the role of hyaluronidase in larval establishment of *H. contortus* infection. In order to block the production of hyaluronidase in infective L3 larvae, we applied the RNAi to L3 larvae. To ascertain that expression of hyaluronidase mRNA was blocked, we detected the mRNA level by real-time PCR. The relative expression profile showed that inhibitory effect was significant (Figure 1A). A standard calibration curve for enzymatic activity was generated that showed a coefficient of determination (R2) 0.999, which indicates that 99.9% of the variation in absorbance can be explained by variation in the concentration (Figure 1B). The calibration curve was represented by y = 0.0013x − 0.0002. The relative enzymatic activity as calculated here also indicated significant decrease in hyaluronidase activity of RNAi treated worms (Figure 1C).

### Effects of hyaluronidase inhibition on worm burden and morphology

**Figure 2:**
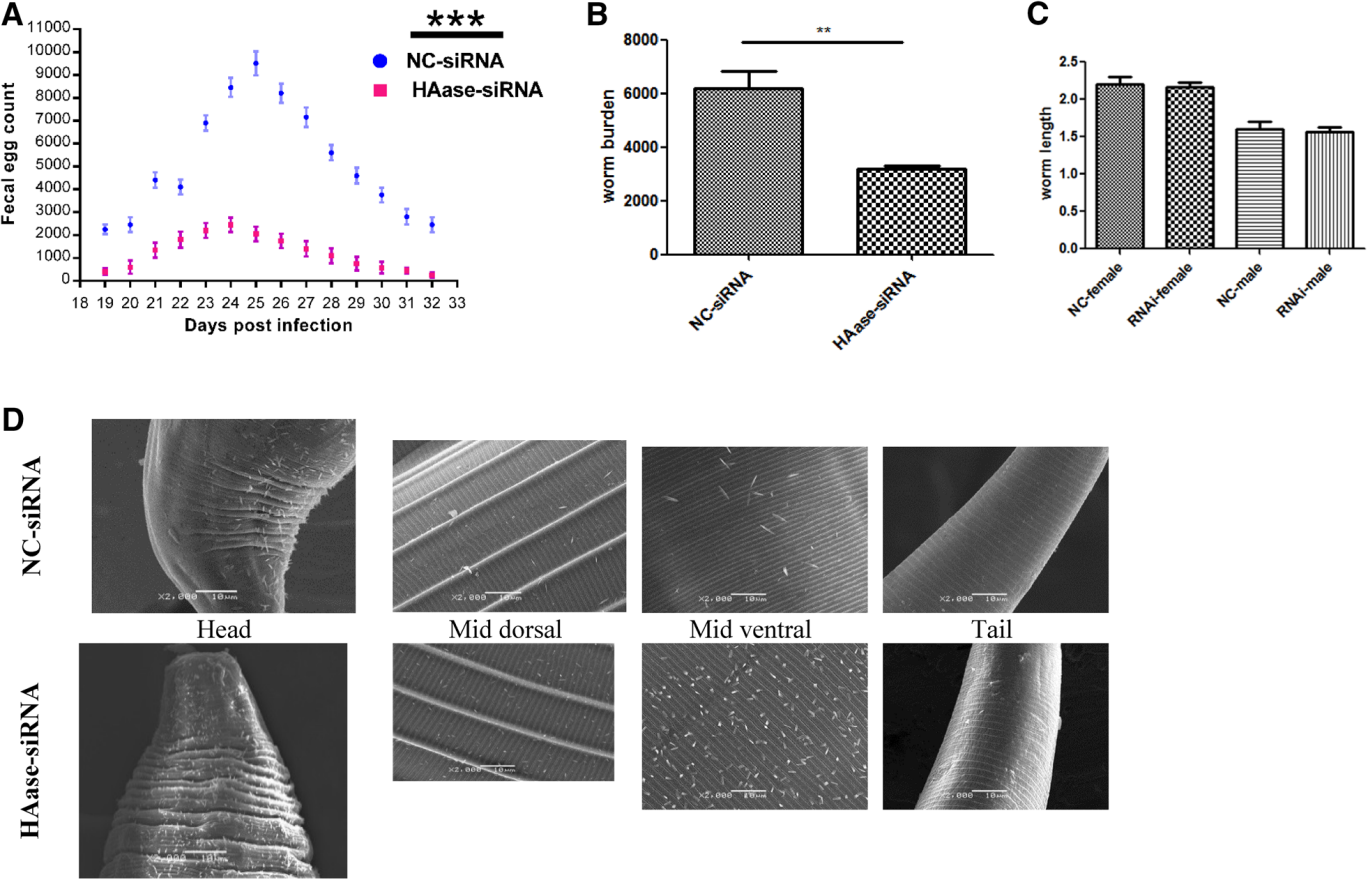
**Worm burden and morphometric analyses of** ***H. contortus*** **at post RNAi treatment**. **A** Fecal Egg Count (during 18 day post infection (dpi) to 32 dpi; **B** Worm burden in sheep; **C** worm length of males and females in both groups; **D** Scanning Electron Microscopy (SEM) observation (×2000) of both groups (Adult worms). Same regions of body are shown in both groups: upper row, NC-siRNA; lower row, HAase-siRNA. No major morphological difference was observed between the two groups. Values represent mean ± SD of five biological replicates. ***p < 0.0001.

After having blocked the hyaluronidase in L3 larvae by RNAi, we observed the effects of hyaluronidase inhibition in larval establishment during the infection. We first analysed the worm burden by fecal egg count. Fecal eggs were counted in both groups after day 18 of infection, and we observed a significant drop in the number of fecal eggs from the RNAi treated group as compared to control group (Figure 2A). After day 33 of infection, sheep were slaughtered and worm burden was analysed by counting the adult worms recovered from abomasa of slaughtered animals. This also showed a significant drop in the worm burden in RNAi treated group against the control group (Figure 2B). To observe morphological differences that likely occurred among the worms of the two groups, we performed morphometric analyses on the adult worms recovered from the abomasa of slaughtered animals. No obvious differences were found in the body lengths of male and female worms in treated group against the control (Figure 2C). We also performed electron microscopy to capture any marked variations in the morphological features of the worms (Figure 2D). We carefully observed the head region, the middle and lower portion of the worm body, and the tail of the worm, in both groups. No significant change was observed in the morphological features of the worms in both groups.

### Effects of hyaluronidase interference on larval invasion of abomasal tissue

**Figure 3:**
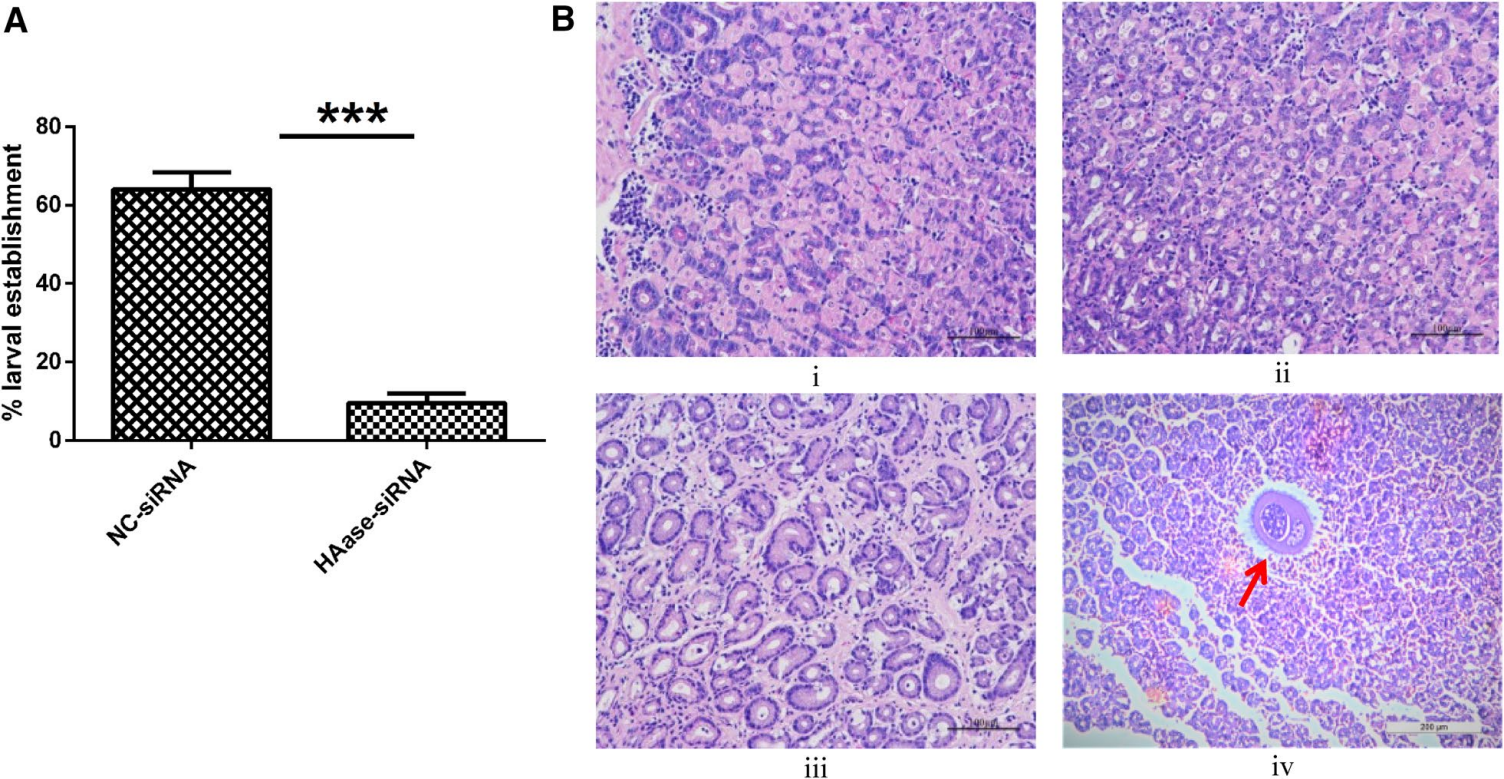
**In vitro analysis of** ***H. contortus*** **infection of sheep abomasal tissue**. Abomasal explants were subjected to L3 larvae penetration of both groups. **A** Tissue larval establishment in both groups. Values represent mean ± SD of five replicates. ***p < 0.0001. **B** H&E sections (×200) of sheep abomasal tissue: i, cross section of normal sheep’s abomasal tissue (control group); ii, cross section of abomasal explant infected by HAase-siRNA treated L3 larvae; iii, cross section of abomasal explant infected by NC-siRNA group L3 larvae; iv, worm penetration can be seen in cross section of abomasal explant infected by NC-siRNA group L3 larvae.

In order to observe the effects of hyaluronidase interference on larval invasion of abomasal tissue, we conducted an in vitro assay of *H. contortus* infection of sheep abomasal tissue (Figure 3). We subjected abomasal explants to L3 larvae penetration of both groups (control and siRNA treated) for 3 h and carefully observed the larval invasion of both groups. Tissue larvae count showed a significant drop in larval invasion in the siRNA treated groups as compared to control group (Figure 3A). To further investigate the role of hyaluronidase in the larval establishment of abomasal tissue, we observed the abomasal surface changes in both groups in comparison to normal sheep’s abomasal tissue by microscopy (Figure 3B). Results showed that the cross sections of tissue infected with siRNA treated group of larvae were much similar in appearance to that of the normal sheep’s abomasal sections. In contrast, the cross sections of explant infected with control group of larvae were uneven and damaged in appearance as compared to that of the normal sheep’s abomasal sections. Furthermore, penetrating worms were also spotted in the cross sections of the explant infected with control group of larvae (Figure 3B, iv). The larval establishment and invasion rate was significantly reduced when hyaluronidase was blocked in the infective larvae.

### Immunohistochemistry showed suppressed level of hyaluronidase in siRNA treated group

**Figure 4:**
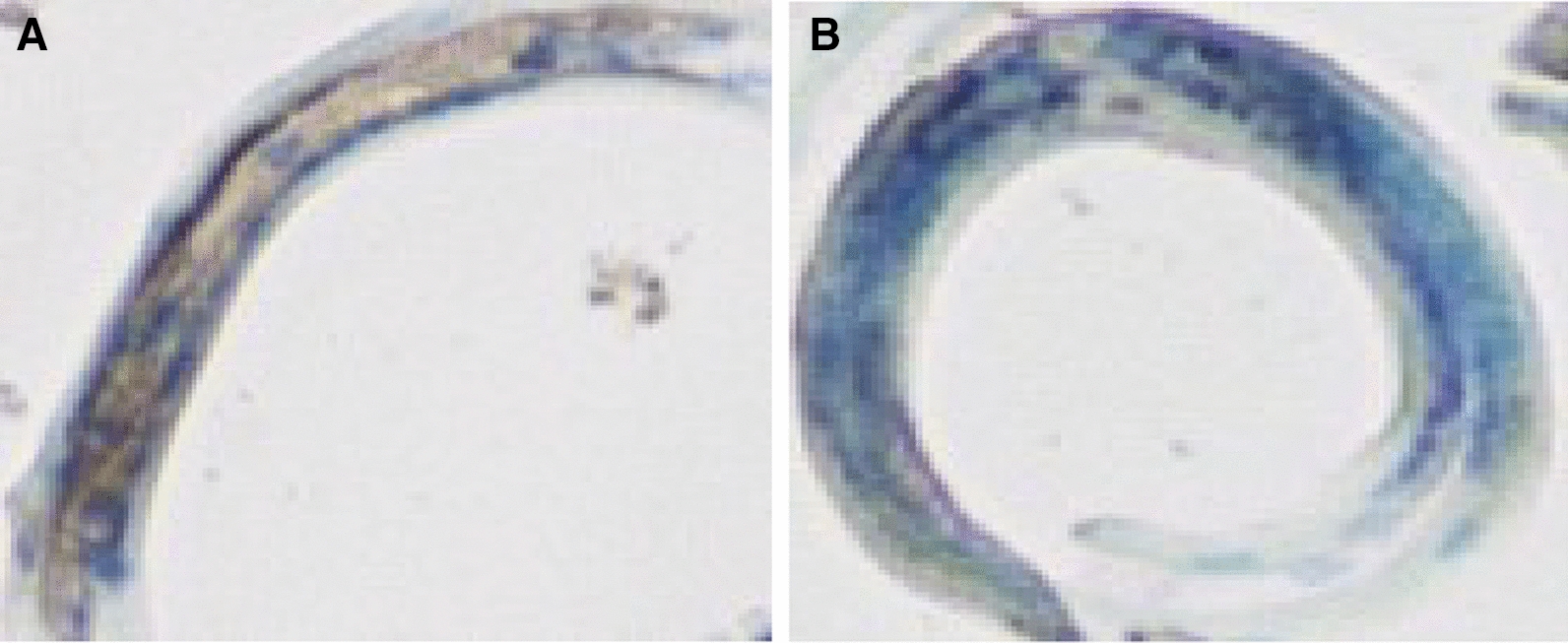
**Immunohistochemistry (×200) to differentiate the Hyaluronidase expression between the two groups.** Immunohistochemistry was performed in the larvae: Anti-hyaluronidase antibody was used to stain (brown) the tissues having hyaluronidase. Harris hematoxylin (blue) was used as counterstain. Brown parts show the presence of hyaluronidase, and parts in blue reflect its absence. In negative control group (**A**) higher expression of Hyaluronidase can be seen. The HAase-siRNA group (**B**) shows a significant lacking of hyaluronidase expression.

Finally we performed the immunohistochemistry to observe the expressed hyaluronidase in L3 larvae (Figure 4). We used the anti-hyaluronidase antibodies to capture the expressed protein in infective larval stage. We observed a high expression level of hyaluronidase in the larvae of control group (Figure 4A–D). Whereas the larvae of siRNA treated group significantly lacked hyaluronidase expression (Figure 4A, B).

## Discussion

During transition from a free-living to a parasitic life style in the life cycle of *H. contortus,* the L3 larvae encounter hyaluronic acid in the ground substance of host’s abomasal tissue. The hyaluronidase released by infective larvae of *H. contortus* [39] would degrade the abomasal mucosa, and could facilitate tissue invasion and larval establishment. In this study we tested this hypothesis and our results showed that hyaluronidase enhanced the larval invasion of the abomasal tissue during *H. contortus* infection. After some preliminary studies on *H. contortus* [26, 45, 46] hyaluronidase was identified as being temporally expressed during L3 to L4 developmental stage in *H. contortus* [39], and it was proposed to play a role associated exclusively with the L3–L4 developmental process. Hyaluronidase has been identified as a virulence factor utilized by the parasitic nematodes *Anisakis simplex*, *Ancylostoma braziliense*, and *A. caninum* while penetrating host’s tissue [37, 38]. Hyaluronidase activity in invasion of hosts’ intestine has also been described previously in intestinal protozoan parasites *Entamoeba histolytica* [47] and *Blastocystis hominis* [48].

We successfully blocked the hyaluronidase gene by RNAi, which was confirmed by qPCR, enzymatic activity, and immunohistochemistry assays. RNAi is an established technique in *H. contortus* [49–51], and previously we and others have successfully applied the RNAi based silencing of target genes in *H. contortus* [21, 22, 52–54]. We investigated the role of hyaluronidase in larval establishment through in vivo assays, which showed a significantly drop in fecal eggs count and worm burden. These findings show that hyaluronidase has a role in the larval establishment of *H. contortus*, and when it was inhibited the worm burden significantly dropped. Whereas, no obvious effects of hyaluronidase were found on worm morphology. To further examine the effects of blocking hyaluronidase, we also applied an ovine ex vivo model [43, 44] where siRNA treated group of larvae showed significantly reduced invasion of the abomasal tissue explants in comparison to control group. Hyaluronic acid is present in the ground substance of abomasal mucosa of the host, whereas the hyaluronidase is expressed during L3 to L4 developmental process in *H. contortus* [39]. Thus, release of hyaluronidase at this stage helps the larvae to dissolve the hyaluronic acid in the abomasal tissue of host, which in turn assists the larvae to invade through and colonize the host’s tissue, and ultimately invading larvae get the protection and access to nutrients. These findings indicate that hyaluronidase plays a key role in host’s tissue invasion and larval establishment, and it is used as a virulence factor by *H. contortus* to invade the host’s tissue. Thus blocking of hyaluronidase gene, and subsequently reduced expression of protein (hyaluronidase) in L3 larvae resulted in an overall significantly reduced level of *H. contortus* infection during the present study. By performing RNAi based gene silencing followed by in vivo and in vitro assays we showed that blocking the expression of hyaluronidase significantly reduced the larval establishment and worm burden, and like others [37, 38, 47, 48] we speculate for *H. contortus* that release of hyaluronidase by its larvae helps them to dissolve the HA in the abomasal tissue of host and facilitate the tissue penetration. However, HA is also present in the extracellular cuticle of larvae, and thus hyaluronidase could also hydrolyse nematode HA during molting as another putative role while establishing the infection [39], which can be analysed in further studies.

In conclusion, we explored the role of hyaluronidase in early establishment of infection by *H. contortus*. Our findings show that hyaluronidase is used as a virulence factor by *H. contortus*, facilitating tissue invasion and larval establishment. These findings provide a new target for the therapeutic strategies to control the infection caused by *H. contortus*.

## Supplementary information

**Additional file 1**. Comparison of Haemonchus contortus’ HAase gene (% amino acid identity) with other nematodes.

**Additional file 2.** Details of siRNA sequences used in present study.

**Additional file 3.** Primers for qPCR assays used in present study.

## Data Availability

All supporting data is presented either within the article or within the additional files of this article.
