# Supplementary material for: Suppression of hyaluronidase reduces invasion and establishment of Haemonchus contortus larvae in sheep
Source: Vet Res. 2020 Aug 27;51:106. doi: 10.1186/s13567-020-00831-8 (PMC7534805; doi:10.1186/s13567-020-00831-8)
Supplement: Supplementary file 1 — Additional file 1. Comparison of Haemonchus contortus’ HAase gene (% amino acid identity) with other nematodes. [file 13567_2020_831_MOESM1_ESM.docx]

**Table S1.** Comparison of Haemonchus contortus’ HAase gene (% amino acid identity) with other nematodes

| **Species** | **Gene** | **%identity with HAase gene of H. contortus** | **Accession** |
| --- | --- | --- | --- |
| Ancylostoma ceylanicum | Hyaluronidase | 66.00% | EPB67756.1 |
| Dictyocaulus viviparous | Hyaluronidase | 63.64% | KJH41058.1 |
| Necator americanus | Hyaluronidase | 64.24% | XP_013296370.1 |
| Oesophagostomum dentatum | Hyaluronidase | 65.72% | KHJ91793.1 |
| Ancylostoma caninum | Hyaluronidase | 62.30% | RCN51930.1 |
| Ancylostoma duodenale | Hyaluronidase | 61.85% | KIH54924.1 |
| Toxocara canis | Hyaluronidase | 46.10% | KHN85392.1 |
| Caenorhabditis elegans | Hyaluronidase | 40.40% | NP_495830.1 |
| Teladorsagia circumcincta | Hyaluronidase | 47.29% | PIO72230.1 |
