# Supplementary material for: Suppression of hyaluronidase reduces invasion and establishment of Haemonchus contortus larvae in sheep
Source: Vet Res. 2020 Aug 27;51:106. doi: 10.1186/s13567-020-00831-8 (PMC7534805; doi:10.1186/s13567-020-00831-8)
Supplement: Supplementary file 2 — Additional file 2. Details of siRNA sequences used in present study. [file 13567_2020_831_MOESM2_ESM.docx]

| **Table S2** Details of siRNA sequences used in present study | | |
| --- | --- | --- |
| **siRNA** | **Sens (5'-3')** | **Antisense (5'-3‘)** |
| HAase-siRNA | GCACGACAUCUGAGGCCAUdTdT | AUGGCCUCAGAUGUCGUGCdTdT |
| NC-siRNA | GCCAAGUCAGCGCUACAGUdTdT | ACUGUAGCGCUGACUUGGCdTdT |
