# Supplementary material for: Suppression of hyaluronidase reduces invasion and establishment of Haemonchus contortus larvae in sheep
Source: Vet Res. 2020 Aug 27;51:106. doi: 10.1186/s13567-020-00831-8 (PMC7534805; doi:10.1186/s13567-020-00831-8)
Supplement: Supplementary file 3 — Additional file 3. Primers for qPCR assays used in present study. [file 13567_2020_831_MOESM3_ESM.docx]

| **Table S3.** Primers for qPCR assays used in present study | | |
| --- | --- | --- |
| **Name** | **Forward (5'-3')** | **Reverse (5'-3‘)** |
| HC-NADH | AAGCGCATGTAGAAGCTCCT | ACGTAAAAACCCTGCAGTTCC |
| HC-HAase | TCCCAACTTGACAGCTTCCC | GCCTAGAAACTCGTGCCCTT |
